# Cationic liposomes induce cytotoxicity in HepG2 via regulation of lipid metabolism based on whole-transcriptome sequencing analysis

**DOI:** 10.1186/s40360-018-0230-5

**Published:** 2018-07-11

**Authors:** Ying Li, Xiu-Liang Cui, Qing-Shan Chen, Jing Yu, Hai Zhang, Jie Gao, Du-Xin Sun, Guo-Qing Zhang

**Affiliations:** 1 Department of Pharmacy, Eastern Hepatobiliary Surgery Hospital, Second Military Medical University, Shanghai, 200438 China; 2 Department of Pharmaceutical Science, College of Pharmacy, University of Michigan, Ann Arbor, MI 48109 USA; 3 National Center for Liver Cancer, Shanghai, 201805 China; 4 The International Cooperation Laboratory on Signal Transduction, Eastern Hepatobiliary Surgery Hospital, Shanghai, 200438 China; 5 Department of Pharmacy, Shanghai First Maternity and Infant Hospital, Tongji University School of Medicine, Shanghai, 201204 China; 6 Department of Pharmaceutical Sciences, Second Military Medical University, 325 Guohe Road, Shanghai, 200433 China

**Keywords:** Cationic liposomes, Nanoparticle, Cytotoxicity, RNA-seq, Lipid metabolism

## Abstract

**Backgroud:**

Cationic liposomes (CLs) can be used as non-viral vectors in gene transfer and drug delivery. However, the underlying molecular mechanism of its cytotoxicity has not been well elucidated yet.

**Methods:**

We herein report a systems biology approach based on whole-transcriptome sequencing coupled with computational method to identify the predominant genes and pathways involved in the cytotoxicity of CLs in HepG2 cell line.

**Results:**

Firstly, we validated the concentration-dependent cytotoxicity of CLs with an IC_50_ of 120 μg/ml in HepG2 exposed for 24 h. Subsequently, we used whole-transcriptome sequencing to identify 220 (77 up- and 143 down-regulated) differentially expressed genes (DEGs). Gene ontology (GO) and pathway analysis showed that these DEGs were mainly related to cholesterol, steroid, lipid biosynthetic and metabolic processes. Additionally, “key regulatory” genes were identified using gene act, pathway act and co-expression network analysis, and expression levels of 11 interested altered genes were confirmed by quantitative real time PCR. Interestingly, no cell cycle arrest was observed through flow cytometry.

**Conclusions:**

These data are expected to provide deep insights into the molecular mechanism of CLs cytotoxicity.

## Background

Gene therapy is a promising approach for the prevention and treatment of severe human diseases such as cancer and AIDs. One of the major obstacles to the clinical success of gene therapy is the lack of effective gene transfer vectors. Offering numerous advantages for gene transfection and drug delivery, nanomaterials are considered as promising diagnostic and therapeutic candidates in medicine [1]. Synthetic vectors, though generally not as efficient as viral vectors, have the potential advantages of being non-immunogenic, versatile and easier to produce. Moreover, synthetic vectors such as cationic liposomes (CLs) can protect drugs from being degraded in the body before they reach their target, and allow for better control over the timing and distribution of drugs to tumor tissue [2]. For example, CLs have a high affinity to the endothelial cells of tumor blood vessels, thus allowing for selective targeting and delivery of paclitaxel to the tumor microenvironment [3]. To date, liposomes have been the most used nanovectors for drug delivery, of which liposomal doxorubicin and amphotericin B received accelerated approval as early as 1995 [4] followed by liposomal daunorubicin in 1996 [5]. More liposomes are currently undergoing advanced clinical trials [6]. Additionally, CLs have also been verified to protect siRNA from RNase degradation efficiently [7]. With more CLs put into clinical use, their cytotoxicity has become a vital concern. CLs present the unique properties of nanoparticles, such as surface and interface effect, small size effect, and quantum size effect, which lead to their specific distribution and accumulation in organs and tissues in vivo. Moreover, due to high concentrations of negatively charged plasma proteins including glycosaminoglycan, glycoprotein and apolipoproteins, CLs could aggregate into micelles and cause cytotoxicity [8]*.* In a word, CLs may exert their effect on cell function through various ways.

The liver is considered to be the main organ of metabolic clearance for most drugs and xenobiotics. Previous studies have shown that nanoparticles are preferentially deposited in the liver under systemic exposure, resulting in prolonged retention within the organ and some instances that are significantly hepatoxic [9, 10]. Therapeutic or toxicological studies showed that when nanoparticles doses overwhelmed the hepatic biodegradation capacity, the excess would accumulate in the organ over a long period of time [11]. In this study, we chose human liver-derived hepatoma cells (HepG2) to carry out our research. Additionally, HepG2 cells have been used for studying delivery and cytotoxicity of CLs [12, 13].

It was reported that oxidative stress, autophagy, and certain physicochemical properties could induce cytotoxicity of nanoparticles. High reactive oxygen species (ROS) induced by nanoparticles may damage cells by peroxidizing lipids, disrupting DNA [14, 15], interfering with signaling functions, and modulating gene transcription [16]. Other studies demonstrated that inhibition of autophagy reversed cell death caused by cationic PAMAM dendrimers, indicating the cytotoxic role of autophagy [17, 18]. It was reported that some nanoparticles caused the unfolding of fibrinogen [19] and elevation of proinflammatory cytokines [20]. However, the CLs biological activities are complicated and mechanism of CLs cytotoxicity remains ambiguous.

Additionally, there were few studies to identify key genes and pathways involved in cytotoxicity of CLs. Spurred on by the development of deep sequencing technology, we are allowed to investigate the mechanisms of CLs cytotoxicity at the whole-transcriptome level. In the present study, we used high-throughput RNA-seq technology to explore the potential mechanism underlying CLs cytotoxicity at whole-transcriptome level by analyzing changes in the global gene expression profile in HepG2 cells after CLs exposure for the first time. The purpose of this study is to obtain transcriptome information of HepG2 cells exposed to CLs and preliminarily investigate molecular mechanism regarding the relationship between levels of genes expression and cytotoxicity of CLs. In this study, differentially expressed genes (DEGs), related gene ontology (GO) and pathways were determined. Subsequently, gene act, pathway act and co-expression network were constructed to further explore the role of the related genes and pathways.

## Methods

### Preparation of CLs

CLs were prepared by using the thin-film dispersion method as described previously with minor modification [21]. Briefly, 27.97 mg 1,2-dioleoyl-3-trimethylammonium-propane (DOTAP) (Avanti Polar Lipids, 890890P, USA), 11 mg cholesterol (Sigma-Aldrich, C8667, USA) and 10.48 mg DSPE-mPEG 2000 (Avanti Polar Lipids, 880120P, USA) in chloroform solutions were mixed in a round-bottomed flask for 45 min-1 h to form dry film. Then the lipid film was rehydrated with 10 ml 10 mM phosphate-buffered saline (PBS, PH 7.4) using a rotary evaporator (Senco, China, Shanghai). After the liposome film hydration in PBS, the liposome dispersion was ultrasonicated for 10 min. Subsequently, CLs were downsized by a high-pressure hand-held extrusion through 400 nm, 200 nm, and 100 nm polycarbonate and polyester membranes (Whatman, UK) at 65 °C using a liposome extruder LF-1 (Avestin, Canada). The obtained CLs in PBS were stored as stock solution at 4 °C.

### Dynamic light scattering (DLS)

Mean diameter, polydispersity index (PDI), and zeta-potential of CLs were performed by using Malvern Zetasizer ZS90 (Malvern instruments Ltd., UK) according to the standard operation protocol. CLs were diluted 1:10 in Milli-QH_2_O, and all the operations were detected after room temperature balance for 10 min. Each assay was performed in three replications.

### Cell viability assay

HepG2 cells (American Type Culture Collection, HB-8065) were cultured in Dulbecco’s Modified Eagle Medium (DMEM, Invitrogen) containing 10% fetal bovine serum (FBS, Hyclone), 100 U/ml penicillin and 100 μg/ml streptomycin (Corning, USA) and maintained at 37 °C in a humidified 5% carbon dioxide (CO_2_) atmosphere. Cell viability was assessed by the Cell Counting Kit-8 (CCK-8) kit (Dojindo, Japan) according to the instruction. 100 μg/ml ZnO nanoparticles (IBU-tec, Germany) were used as a positive control. Briefly, HepG2 cells were seeded in 96-well plate at a density of 3000/well and incubated for 24 h. The cells were then exposed to a series of concentrations of CLs (25, 50, 100, 200, 400, 800, 1600, 3200 μg/ml) for another 24 h. Added 10 μl of the CCK-8 solution to each well of the plate, incubated the plate for 2 h in the incubator, and measured the absorbance the 450 nm using a microplate reader (Thermo Scientific Multiskan MK3). The data was normalized to the blank, and six independent experiments were conducted. Half-maximum inhibitory concentration (IC_50_) value of CLs was determined by GraphPad Prism 5.

### RNA extraction and RNA-sequencing (RNA-seq)

Total RNA was extracted using Trizol Reagent (Invitrogen, USA) according to the manufacturer’s instruction. RNA quantity and RNA integrity number (RIN) were assessed by Agilent 2200 bioanalyzer. Sample RNA with the RIN value below 6.4 was discarded [22]. The complementary DNA (cDNA) libraries for single-end sequencing were prepared using Ion Total RNA-seq Kit v2.0 (Life technologies, USA) according to the protocol provided by the manufacturer.

### Filtering raw reads and mapping statistics

We applied Fast-QC (http://www.bioinformatics.babraham.ac.uk/projects/fastqc/) software to assess the quality of data, including the distribution of the base quality value, content of GC, proportion of PCR duplication, and frequency of kmers. MapSplice v2.1.8 software was used for RNA-seq read mapping analysis, whose core program is Bowtie. It has been approved a highly accurate algorithm for the alignment of RNA-seq reads to splice junctions [23]. We calculated the mapping rate for filtered clean reads and the distribution of genes on chromosomes.

### Identification of differentially expressed genes (DEGs)

To quantify the expression levels of the transcripts, the RNA-seq data normalization was carried out to obtain an RPKM value [24]. We applied EBSeq to filter the DEGs. After the statistical analysis, we screened the DEGs by the following criteria: fold change>1.50 or fold change<0.67, FDR<0.05.

### Functional analysis of DEGs

#### Gene ontology (GO) analysis

The enriched biological processes were identified using Fisher’s exact test and χ^2^ test, and the false discovery rate (FDR) was calculated to correct the *P*-value. Within the significant category, the enrichment Re was given by Re = (*n*_*f*_/*n*)/(*N*_*f*_/*N*), where *n*_*f*_ is the number of DEGs within the particular category, *n* is the total number of genes with the same category, *N*_*f*_ is the number of DEGs, and *N* is the total number of genes. Only categories that had a significance of *P*-value< 0.05 and FDR<0.05 were reported. GO terms were organized as a directed acyclic graph (DAG), and we performed GO tree analysis to investigate the internal relationship between the enriched GO terms.

#### Pathway analysis

Fisher’s exact test and χ^2^ test were used to identify the significantly enriched pathways of the DEGs, and the threshold of significance was defined by P-value and FDR [25]. Pathway categories with P-value<0.05 and FDR<0.05 were reported.

### Network analysis of DEGs

Network analysis was performed to trace the interactions among DEGs (*p*-value<0.01). Gene interaction and co-expression networks were constructed, and Cytoscape was used for graphical representation [26]. The gene interaction network was built using KEGG pathways as a background network, which provided information about the relationship among the genes, proteins and compounds [27, 28].

The co-expression network was built according to the normalized signal intensity of their expression levels. For each pair of genes, we calculated the Pearson’s correlation and chose the significant correlation pairs to construct the network [29]. Degree centrality and K-core were two widely used topological importance indicators [30, 31]. In our analysis, “key regulatory” genes were determined by the degree and k-core differences between control and CLs-treated groups.

### Quantitative real-time reverse transcription-PCR (qRT-PCR) assay

A 2-step method for qRT-PCR was used to determine mRNA expression level. Briefly, cDNA was synthesized using oligo (dT) primers with PrimeScript™ II 1st strand cDNA synthesis Kit (Takara Biotechnology, China) according to the manufacturer’s protocol. qRT-PCR was performed using Applied Biosystems StepOne™ Real-Time PCR System (Applied Biosystems, USA) and SYBR Premix Ex Taq™ (Tli RNaseH Plus) kit (Takara Biotechnology, China). The primers of these detected genes were shown in Table 5 and primers for endogenous reference gene GAPDH applied a commercial product (Sangon Biotech, China). The expression levels were measured in terms of the cycle threshold (Ct) and were normalized to GAPDH expression using the 2^-△△Ct^ method [32].

### Cell cycle analysis

Cell cycle was analyzed using flow cytometry (FCM). The concentration of 120 μM and exposure of 24 h was applied in CLs-treated group and 25 μM resveratrol was adopted as a positive control group. HepG2 cells were harvested, washed once in PBS, and then fixed in 75% pre-cold ethanol for 6 h at 4 °C. Staining for DNA content was performed with 50 μg/ml propidium iodide (PI) and 1 mg/ml RNase (BD Biosciences, USA) for 15 min at room temperature. Stained cells were analyzed by cell cycle distribution on MACS Quant Analyzer (Germany).

## Results

### Synthesis, characterization and concentration-dependent cytotoxicity of CLs

CLs were successfully synthesized using the thin-film dispersion method. Subsequently, CLs were characterized using DLS to determine their mean diameter and size distribution. All the data represented the mean of three independent experiments. DLS revealed that their mean diameter size was 158 nm and they were well-distributed (PDI = 0.13). The zeta potential of CLs was + 22.1 mV.

**Fig. 1:**
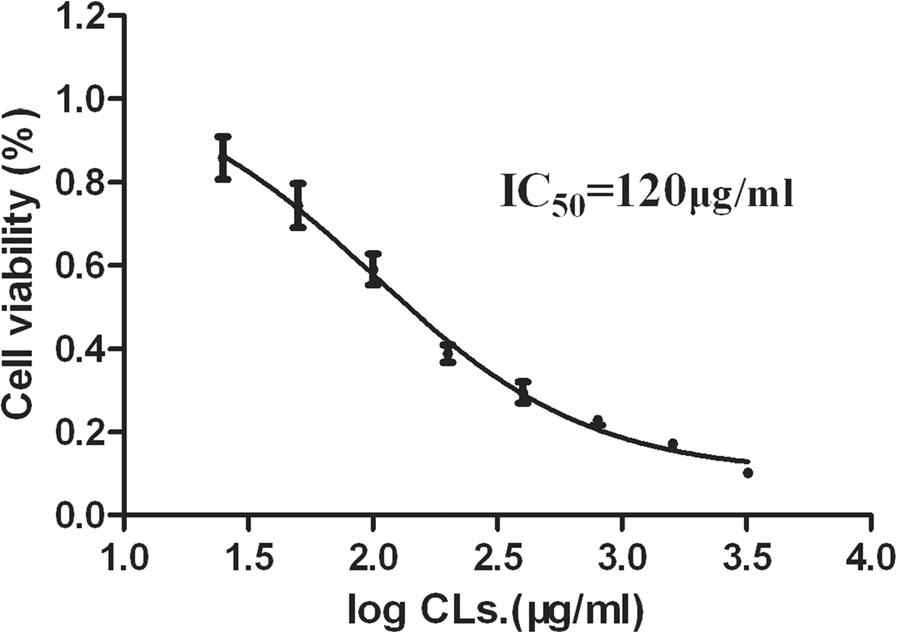
HepG2 cytotoxicity of CLs at a series of concentrations (exposure for 24 h)

In CCK-8 test, morphological alteration was observed under the microscope. Then, the concentration-dependency of cytotoxicity was investigated with an IC_50_ of 120 μg/ml via a cell viability assay, as shown in Fig. 1. It showed 14% inhibition of cell viability and no obvious cytotoxicity at the concentration of 25 μg/ml. However, at the highest concentration (3200 μg/ml), the inhibition increased to approximately 90% after 24 h of CLs exposure, suggesting that the high dose of CLs showed significant cell toxicity. In summary, CLs could induce obvious morphological alteration and significant cell viability reduction in HepG2 cells, which indicated the cytotoxic effect of CLs.

### DEGs identification between control and CLs-treated groups

RNA-seq was employed to investigate the potential mechanism of cytotoxicity of CLs. The concentrations of 6 extracted RNAs were between 0.65–1.22 mg/ml and they had an average RIN value of 8.0 ± 0.6 (average ± standard error). The RNA sequencing generated approximately 12 gigabases (Gb) of raw data that were uploaded in NCBI with GEO accession number GSE89701. The filtered data set comprised approximately 15 million total reads that were distributed almost evenly between the six samples (Table 1), yielding a high sequencing depth (14.71–19.16 million raw data reads per sample). The average GC content was approximately 51% for each sample. Approximately 13.60 ± 1.17 million reads (83.4–87.3%) were mapped to the human genome sequence in the 6 independent samples and 13.00 ± 1.08 million reads were uniquely aligned to the human genome. The RNA-seq data normalization was carried out to determine the RPKM value [24].

**Table 1:** Mapping statistics, reads distribution and quantification of RNA-seq

Sample ID	Raw reads (million)	Unmapped reads (million)	Mapped reads (million)	Mapped ratio (%)	Uniquely mapped ratio (%)
Control-1	15.20	1.94	13.26	87.2	83.5
Control-2	15.25	2.09	13.16	86.3	82.7
Control-3	15.15	1.93	13.23	87.3	83.5
CLs-1	19.16	3.18	15.98	83.4	79.2
CLs-2	15.11	1.98	13.13	86.9	83.0
CLs-3	14.72	1.87	12.85	87.3	83.5

**Fig. 2:**
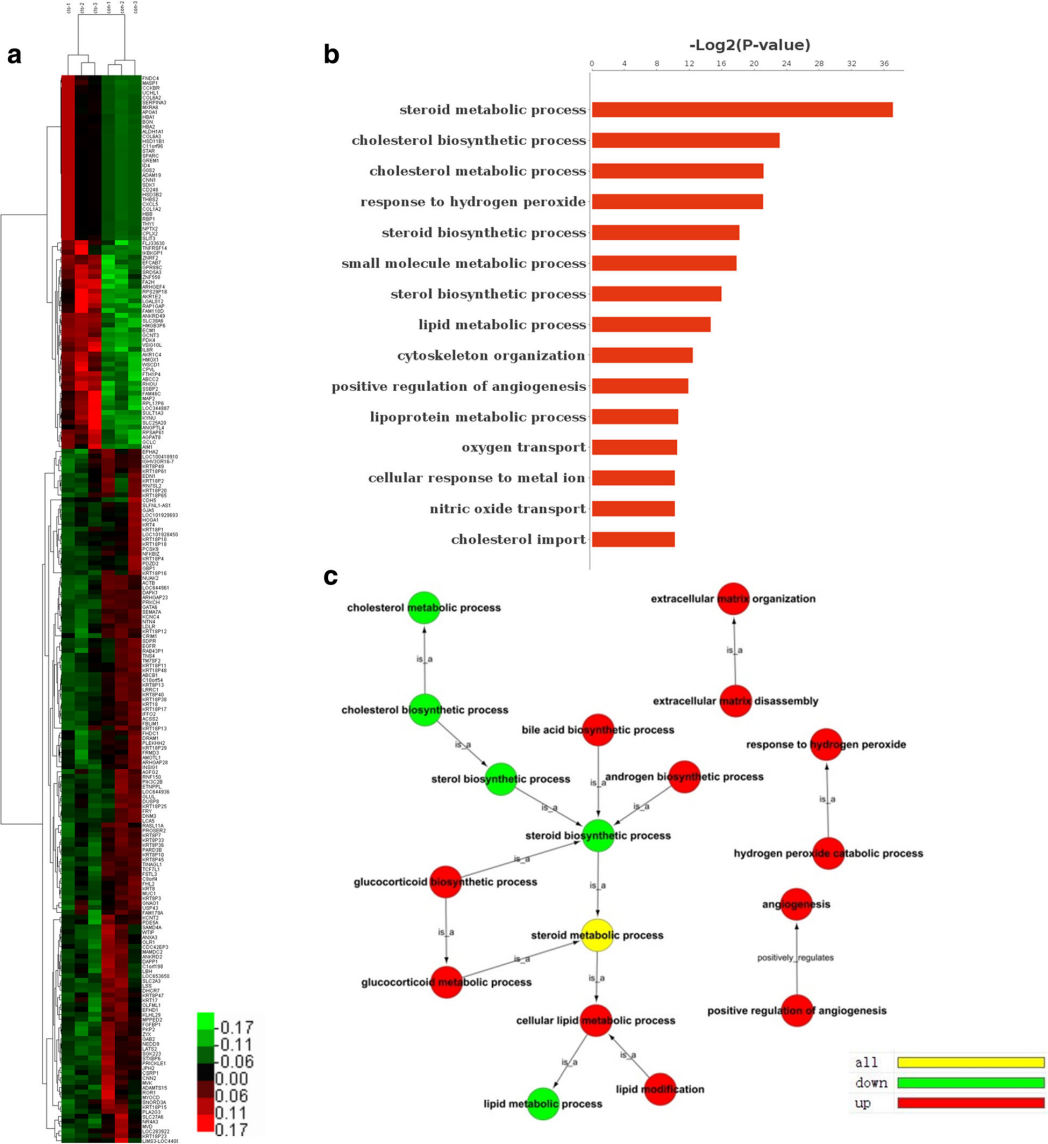
**a** Expression profile heat map of DEGs in HepG2 cells following exposure to CLs. A total of 77 up-regulated and 143 down-regulated genes are shown. **b** GO analysis of DEGs. **c** GO tree analysis of enriched GO terms. Red circles represent up-regulated genes; Green circles represent down-regulated genes; Yellow circles represent ambiguous-regulated genes

To characterize the gene expression changes induced by CLs, the EBSeq algorithm was conducted and the criteria of screening DEGs was as follows: fold change>1.50 or fold change<0.67, FDR<0.05. There were 77 genes up-regulated and 143 genes down-regulated (Fig. 2a). Some of the DEGs were involved in primary and secondary metabolism including PLA2G3, SLC27A6, HOGA1, TM7SF2, DHCR7, LSS, SRD5A3, AKR1C4 and PDK4 and 6 among these were involved in the lipid metabolic process. Besides, there were 4 DEGs including NFKBIZ, DRAM1, DAPK1 and HMOX, respectively involved in inflammatory, autophagy, cell death, and antioxidant response (Table 2).

**Table 2:** Gene regulated by cationic liposomes in the HepG2 cell

Gene ID	Gene Symbol	Description	Log_2_FC	FDR
50,487	PLA2G3	Group 3 secretory phospholipase A2	−3.02	2.88E-02
28,965	SLC27A6	cDNA, FLJ94000, highly similar to *Homo sapiens* solute carrier family 27 (fatty acid transporter), member 6 (SLC27A6), mRNA	−1.47	7.5E-03
112,817	HOGA1	4-hydroxy-2-oxoglutarate aldolase, mitochondrial	−1.23	3.6E-03
1612	DAPK1	Death-associated protein kinase beta	−1.20	0
64,332	NFKBIZ	NF-kappa-B inhibitor zeta	−1.04	1E-05
7108	TM7SF2	Transmembrane 7 superfamily member 2, isoform CRA_a	−0.95	9.9E-03
1717	DHCR7	7-dehydrocholesterol reductase, isoform CRA_a	−0.82	5.18E-11
4047	LSS	cDNA, FLJ92849, highly similar to Homo sapiens lanosterol synthase (2,3-oxidosqualene-lanosterolcyclase) (LSS), mRNA	−0.66	1.59E-05
55,332	DRAM1	DNA damage-regulated autophagy modulator protein 1	−0.60	4.44E-02
79,644	SRD5A3	Polyprenolreductase	0.75	2.75E-04
1109	AKR1C4	Aldo-ketoreductase family 1 member C4	1.00	6.59E-07
3162	HMOX1	Heme oxygenase 1	1.21	3.12E-03
5166	PDK4	Pyruvate dehydrogenase kinase, isoenzyme 4	2.54	0

### GO and pathway analysis

Of these DEGs, 61 up- and 88 down-regulated, respectively, could be annotated with gene ontology categories. GO analysis indicated that 10 GO terms were enriched (*P* < 0.05, FDR < 0.05). DEGs were mainly involved in multiple metabolic biological processes, including steroid metabolic process, cholesterol biosynthetic process, cholesterol metabolic process, steroid biosynthetic process, small molecule metabolic process, sterol biosynthetic process, and lipid metabolic process (Fig. 2b). Notably, the GO category for “steroid metabolic process” encompassing 15 genes (p<10^− 12^) was the most strongly changed category, and Hydroxy-delta-5-steroid dehydrogenase gene HSD3B2, Corticosteroid 11-beta-dehydrogenase isozyme 1 gene HSD11B1 and Steroidogenic acute regulatory protein gene STAR were among the most affected genes in this category. Among the 149 DEGs, there were 34 genes participating in the small molecule metabolic process (Table 3). In addition to the above categories, we found other GO terms are also involved in response to hydrogen peroxide, cytoskeleton organization and positive regulation of angiogenesis (Fig. 2b). GO tree can help us to make certain the relationship of the enriched GO terms. As shown in Fig. 2c, the biosynthetic process of cholesterol and steroid is in the term of their metabolic process, respectively. In addition, the term “cholesterol biosynthetic process” is in the term “steroid biosynthetic process” which is included in the most significant category “steroid metabolic process” and all the above are in the category “lipid metabolic process”. Down-regulation of genes mainly occurs under the GO terms of cholesterol biosynthetic process, cholesterol metabolic process, sterol biosynthetic process, steroid biosynthetic process, and lipid metabolic process, while a majority of other terms consist of up-regulated genes. Taken together, we showed that CLs altered genes that were strongly correlated to the lipid metabolic process.

**Table 3:** Gene Ontology (GO) categories of differentially expressed genes (DEGs)

GO ID	Term	Total	Significant	Genes	P-value
GO: 0008202	steroid metabolic process	122	15	INSIG1, DHCR7, AKR1C4, SRD5A3, PCSK9, MVD, APOA1, LDLR, MVK, HSD3B2, HSD11B1, STAR, TM7SF2, LSS, SULT1A3	6.79E-12
GO: 0006695	cholesterol biosynthetic process	34	7	INSIG1, DHCR7, MVD, APOA1, MVK, TM7SF2, LSS	1.08E-07
GO: 0008203	cholesterol metabolic process	89	9	INSIG1, DHCR7, PCSK9, MVD, APOA1, LDLR, MVK, STAR, TM7SF2	4.28E-07
GO: 0042542	response to hydrogen peroxide	43	7	OLR1, GNAO1, HMOX1, HBA1, HBB, STAR,	4.43E-07
GO: 0006694	steroid biosynthetic process	60	7	DHCR7, MVD, MVK, HSD3B2, STAR, TM7SF2, LSS	3.35E-06
GO: 0044281	small molecule metabolic process	1410	34	ANGPTL4, PIK3C2B, ABCB1, INSIG1, FHL2, KYNU, ALDH1A1, DHCR7, HMOX1, HBA2, HBA1, PLA2G3, GPAT3, ACSS2, AKR1C4, HBB, SRD5A3, GLUL, SLC25A20, MVD, PDK4, APOA1, G0S2, LDLR, MVK, HSD3B2, GCLC, BGN, SLC2A3, HSD11B1, STAR, TM7SF2, LSS, SULT1A3	4.28E-06
GO: 0016126	sterol biosynthetic process	29	5	INSIG1, DHCR7, MVD, MVK, TM7SF2,	1.55E-05
GO: 0006629	lipid metabolic process	490	16	INSIG1, DHCR7, FA2H, PLA2G3, AGPAT9, SRD5A3, PCSK9, MVD, APOA1, LDLR, SLC27A6, MVK, HSD11B1, TM7SF2, LSS, SULT1A3	3.91E-05
GO: 0007010	cytoskeleton organization	7	117	KRT4, NEDD9, THY1, WTIP, RHOU, KRT8, CNN2	1.80E-04
GO: 0045766	positive regulation of angiogenesis	6	88	ANGPTL4, ECM1, HMOX1, GREM1, GATA6, ANXA3	2.65E-04

**Fig. 3:**
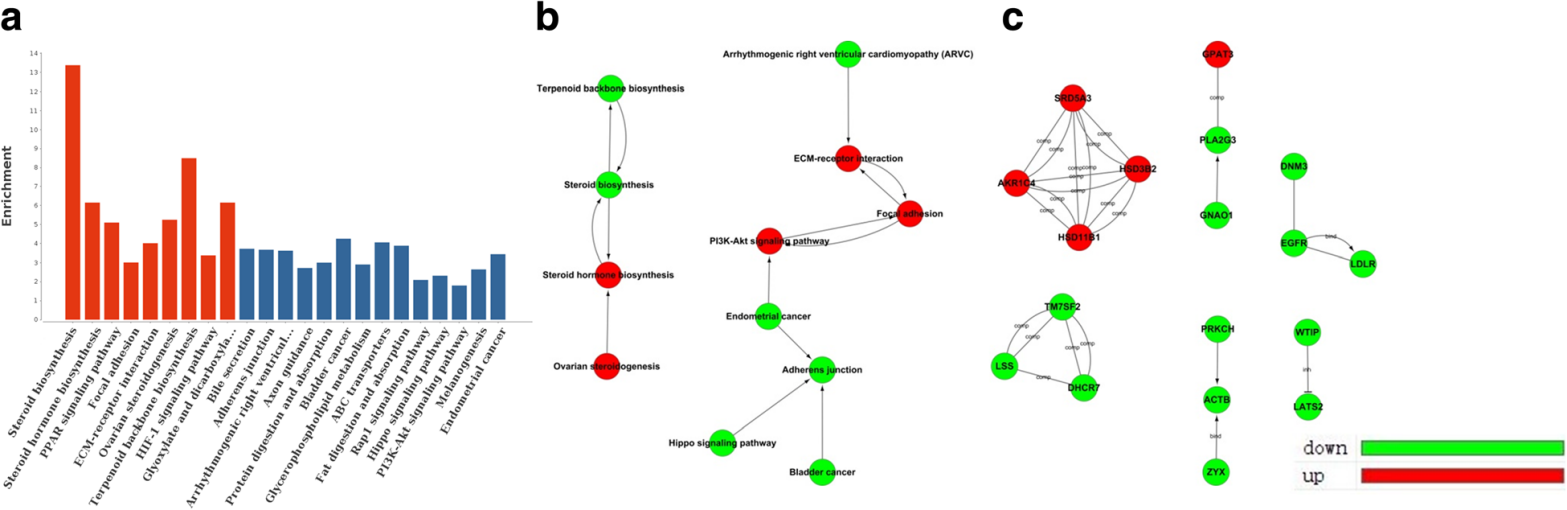
**a** Pathway analysis of DEGs. Red represents the significant pathway. **b** Pathway act network. **c** Gene act network

The pathway analysis showed the DEGs were associated with steroid biosynthesis, steroid hormone biosynthesis, PPAR signaling pathway, focal adhesion, ECM-receptor interaction, ovarian steroidogenesis, Terpenoid backbone biosynthesis, HIF-1 signaling pathway and glyoxylate, dicarboxylate metabolism (*P* < 0.05) (Fig. 3a). Among them, 5 pathways were involved in metabolism. We built the pathways act network to perform deep analysis (Fig. 3b). It was obvious that steroid biosynthesis and focal adhesion followed by steroid hormone biosynthesis, ECM-receptor interaction and PI3K-Akt signal pathway were the most important pathways involved, and these pathways were located at the centers of each cluster and showed more interactions with their surrounding pathways.

### Network analysis

To illustrate the biological effect of CLs, we built the network of DEGs using the KEGG database. An important network module including HSB11B1, AKR1C4, SRD5A3 and HSD3B2 was identified (Fig. 3c). Interestingly, these genes were all involved in the most significant GO category steroid metabolic process.

**Fig. 4:**
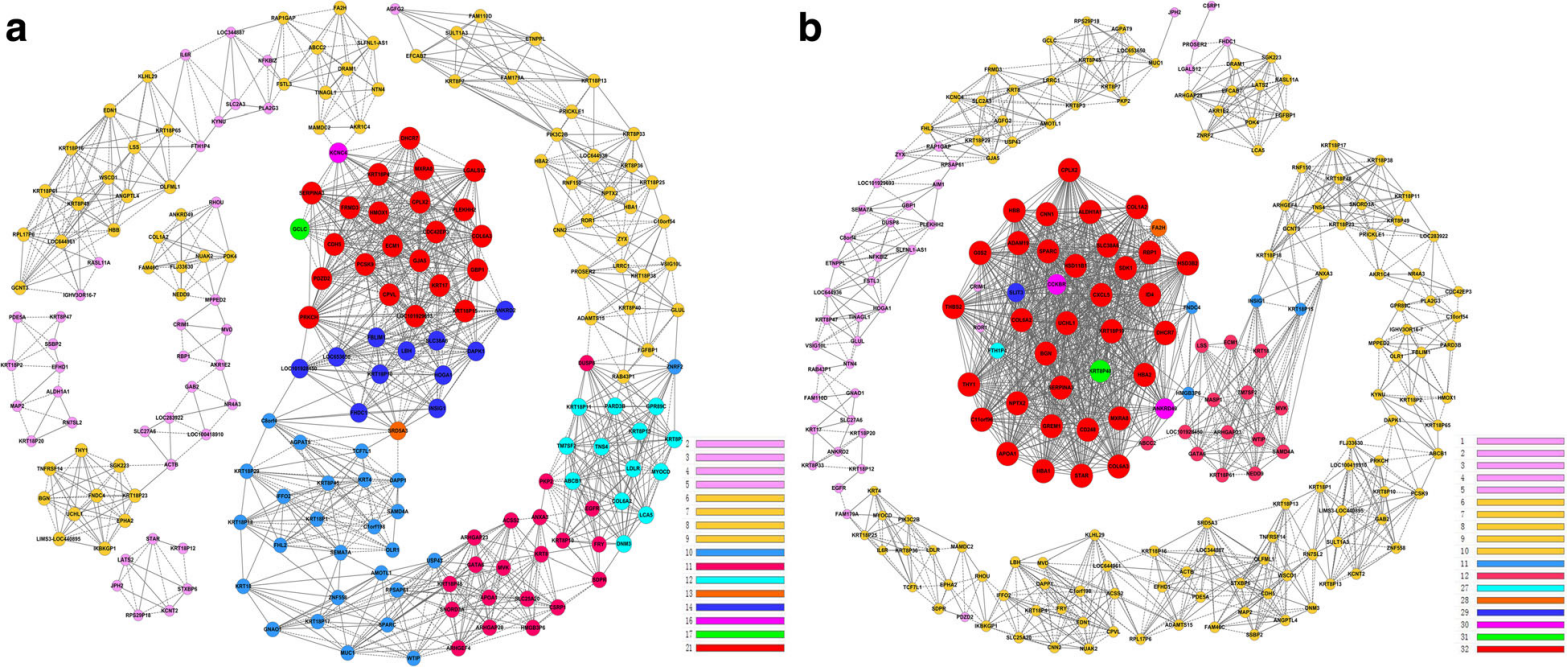
Co-expression network of DEGs in the control (**a**) and CLs-treated (**b**) groups. Solid lines represent positive correlation, and dashed lines represent negatively correlation. The size and color of the nodes correspond to their co-expression ability. The greater the size of the node, the greater the number of its direct neighbors

In co-expression network analysis, “key regulatory” factors were determined by the degree and k-core differences between the control and CLs-treated groups. As shown in Fig. 4 and Table 4, HSD11B1, HSD3B2, G0S2, and CXCL5 followed STAR possessed the biggest degree differences. HSD11B1, HSD3B2, STAR, FA2H and DHCR7 were members of steroid metabolic process. G0/G1 switch gene 2 (G0S2) is an important negative regulator of the rate-limiting lipolytic enzyme adipose triglyceride lipase-mediated lipolysis [33]. Chemokine C-X-C motif ligand 5 (CXCL5) is manifested to participate in the inflammatory process for nanotoxicology [34].

**Table 4:** Intersection of DEGs between control and CLs-treated groups

Gene	Degree		K-core		Dif Degree	Dif K-core
	control	CLs-treated	control	CLs-treated		
G0S2	0	38	0	32	38	32
HSD11B1	0	38	0	32	38	32
HSD3B2	0	38	0	32	38	32
CXCL5	0	38	0	32	38	32
STAR	6	37	4	32	31	28
FA2H	9	31	7	28	22	21
DHCR7	23	38	21	32	15	11

### qRT-PCR and cell cycle analysis

**Fig. 5:**
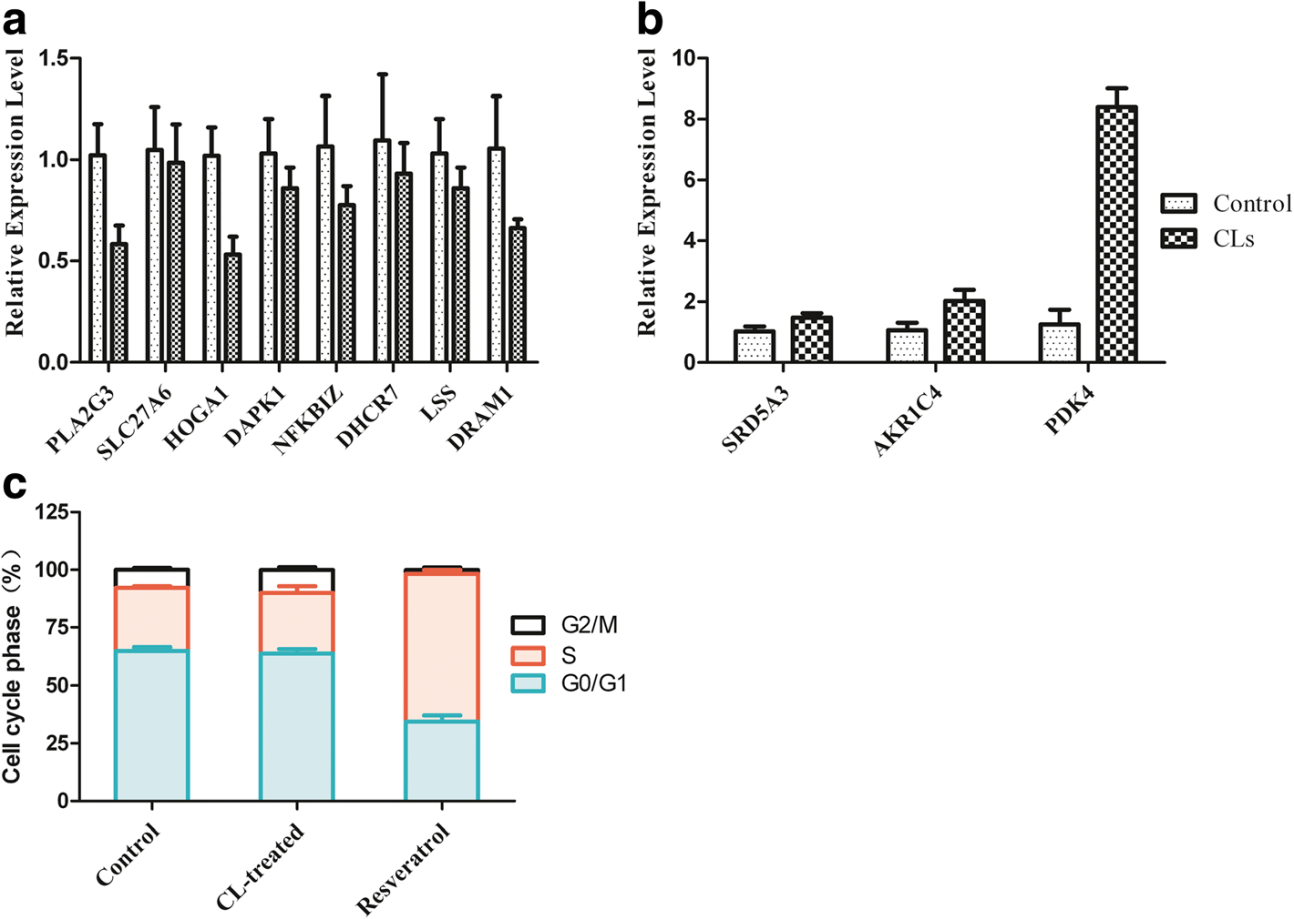
**a**, **b** qRT-PCR verification of selected DEGs including 8 down-regulated and 3 up-regulated genes. The relative expression levels of these genes were normalized to GAPDH. **c** HepG2 cell cycle analysis was performed by flow cytometry. The percent of cells in each phase of the cell cycle was shown

qRT-PCR was performed to validate the relative gene expression of the 11 selected genes. As shown in Fig. 5a and b, the mRNA expression levels of 8 up-regulated and 3 down-regulated genes measured by qRT-PCR were almost comparable to the RNA-seq results. We analyzed the fold change of the gene expression ratios between RNA-seq and qRT-PCR by linear regression, the overall correlation coefficient was 0.9808, indicating the reliability of the RNA-seq data.

FCM analysis was applied to detect whether cell cycle arrest was induced. Resveratrol is well known as a positive compound to trigger accumulation of cells in S-phase of the cell cycle. As shown in Fig. 5c, no significant differences in cell cycle progression could be observed between control and CLs-treated groups. However, resveratrol triggered retention of HepG2 cells in S-phase, showing that these cells were not resistant to cell cycle arrest (Fig. 5c).

## Discussion

Using whole-transcriptome sequencing and computational approaches, we showed herein that CLs caused changes in gene expression in HepG2 cells and that gene categories related to lipid metabolism were the most significantly affected categories. In the previous studies, CLs were reported to interact with negatively charged cellular components (opsonin, serum protein and enzyme) resulting in hemolysis, impairment of mitochondrial function and membrane integrity in vitro [12, 35]. In vivo, hepatotoxicity and weight loss have also been observed in mice after systemic administration of cationic siRNA nanoparticles [36]. In our study, the cytotoxicity of CLs in HepG2 was studied with a CCK-8 test after 24 h exposure. Morphological alteration under the microscope and concentration-dependency of cytotoxicity with IC_50_ of 120 μg/ml via a cell viability assay indicated the cytotoxic effect of CLs. To more clearly and exactly identify the changed level of DEGs, the concentration of IC_50_ (120 μg/ml) and exposure time of 24 h were chosen for subsequent experiments.

In the transcriptome sequencing, there were several DEGs involved in primary and secondary metabolism. Remarkably, 6 DEGs including PLA2G3, SLC27A6, TM7SF2, DHCR7, LSS and SRD5A3 were involved in the lipid metabolic process (Table 2). In addition, in the down-regulated genes, NFKBIZ, NF-kappa-B inhibitor zeta, is a member of the ankyrin-repeat family. The C-terminal portion of the encoded product shares high sequence similarity with the I kappa B family of proteins. The latter are known to play a role in inflammatory responses. DRAM1, DNA damage regulated autophagy modulator 1, was reported to increase autophagy flux through promoting lysosomal acidification and protease activation [37]. It was found that DRAM1 knockdown inhibited autophagy flux and aggravated cell injury in Neuro-2a cells [38]. A recent study has shown that CLs induced cell necrosis involved in late-stage autophagic flux inhibition [39]. DAPK1, death-associated protein kinase 1, is an important regulator of cell death and autophagy [40]. Additionally, it is a mediator of pro-apoptotic pathways and is involved in multiple cell death processes induced by various internal and external apoptotic stimulants [41]. In the up-regulated genes, HMOX1, Hemeoxygenase 1, is a gene for the antioxidant response and is considered to be a marker of oxidative stress. In a previous study, the expression of HMOX1 was increased in liver tissue after 7 days of repeated doses of CLs in rat [42]. In summary, apart from cellular metabolism process, the DEGs were also involved in inflammatory, autophagy, cell death, and antioxidant response.

In GO enriched terms, DEGs were mainly involved in multiple metabolic biological processes, including steroid metabolic process, cholesterol biosynthetic process, cholesterol metabolic process, steroid biosynthetic process, small molecule metabolic process, sterol biosynthetic process, and lipid metabolic process (Table 3). Moreover, to make certain the relationship of the enriched GO terms, we constructed the GO tree graph. The biosynthetic process of cholesterol and steroid is in the term of their metabolic process, respectively. In addition, the term “cholesterol biosynthetic process” is in the term “steroid biosynthetic process” which is included in the most significant category “steroid metabolic process” and all the above are in the category “lipid metabolic process” (Fig. 2c). At present, it is well accepted that cellular energy metabolism disturbance is one of the most important mediators of disease occurrence. Dysregulation of cellular energy is one of the hallmarks of cancer, and metabolic reprogramming is attracting increased attention in cancer research [43]. Many mechanisms for cytotoxicity of nanoparticles have been explained but little has been reported on the energy metabolism response. A previous study has shown that excessive exposure to certain metal nanoparticles can cause cellular metabolic turbulence [44]. Lipid metabolism is an important resource for cellular energy. In our study, GO results revealed that cytotoxicity of CLs mainly correlates with lipid metabolism, in addition to a response to hydrogen peroxide and dysfunction of angiogenesis.

In pathway analysis, 5 of the enriched 9 pathways including steroid biosynthesis, steroid hormone biosynthesis, Glyoxylate and dicarboxylate metabolism were involved in metabolism. Additionally, the PPARs are a group of nuclear receptors that are activated by fatty acids and their derivatives. Each of the three distinct subtypes is encoded by a separate gene and binds fatty acids and eicosanoids. Their activation leads to interruption of cell metabolism, cell growth and stress response [45]. Some reports suggested PPAR modulation by either agonist or antagonist might be a potential treatment for metabolic diseases [46]. Focal adhesion, transmembrane junctions between the extracellular matrix and the cytoskeleton, consists of a large number of both cytoskeletal and signal transduction (adapter) proteins and are rich in tyrosine phosphorylated proteins [47]. In cell biology, focal adhesions are large macromolecular assemblies that serve as the mechanical linkages to the ECM and as a biochemical-signaling hub to concentrate and direct numerous signaling proteins. Activation of the PI3K/AKT signaling pathway is implicated in the regulation of cell proliferation, death and metastasis [48]. Pathway results indicated that down-regulation of steroid biosynthesis and dysfunction of focal adhesion through PI3K/AKT signaling may be the key biological events after CLs exposure and the dominant elements involved in cytotoxicity in HepG2. Combining the results of GO and pathway analysis, we concluded that during the process of CLs entering into the cells, HepG2 cells produced a series of cellular responses such as a disorder of energy metabolism, regulation of microvascular function, dysfunction of focal adhesion and response to oxidative stress. Collectively, steroid biosynthesis was highly correlated to cytotoxicity induced by CLs.

To explore the potential relationship between the 220 DEGs involved in CLs cytotoxicity, we constructed co-expression networks for the control group and CLs-treated group. The key genes HSD11B1, HSD3B2, STAR, FA2H and DHCR7 indicated cytotoxicity of CLs mainly related to lipid metabolism. Previous studies have suggested that the size, shape and surface charge could affect the therapeutic effect and cytotoxicity of nanoparticles [49, 50]. Our results revealed the toxicity of CLs in HepG2 was mainly related to lipid metabolic process, which was possibly because of the materials of the nanoparticles. It indicates that in addition to the size and surface charge, the materials of nanoparticles may play an important role in their cytotoxicity and further studies are needed to conclusively explain their toxicity mechanism.

To confirm the accuracy and reproducibility of the transcriptome analysis results, 11 genes were selected from the list in Table 5 in our further study, and qRT-PCR was performed to validate the relative gene expression of these genes. Of these, 8 genes were in the following GO categories: steroid metabolic process, cholesterol biosynthetic process, cholesterol metabolic process, steroid biosynthetic process, and lipid metabolic process. 2 genes including DAPK1 and DRAM1 are related to apoptosis and autophagy. NFKBIZ is a well-known factor associated with the inflammatory response.

**Table 5:** Primer sequences for quantitative Real-time reverse transcription-PCR (qRT-PCR) assay

Gene	Primer sequences (5′-3′)
PLA2G3	Forward:AGAGAGGATGGACCATGCCT Reverse:GTTCCCGGCAACAGAGATCA
SLC27A6	Forward: TCCTGTGGGCTTTTGGTTGT Reverse: AAGTGGCACCCAACTCAACA
HOGA1	Forward: GGATCCCAGGGCTGAAGAAA Reverse: CTGGTGAAATCCATGCGCAG
DAPK1	Forward: AAGATCAAGTGCTGCCTGCT Reverse: GGCTGGTAGATCATGACGGG
NFKBIZ	Forward: GCCCAGTTGCCTGTCTTTTG Reverse: TTCCTCATCAACAGGCGGAC
DHCR7	Forward: CCAGGTGCTTCTGTACACGT Reverse: ACTTGTTCACAACCCCTGCA
LSS	Forward: GAGCGGCGTTATTTGCAGAG Reverse: AGACACCGGACTCCTCTCTC
DRAM1	Forward: CATCTCTGCCGTTTCTTGCG Reverse: AAACCAAAGGCCACTGTCCA
SRD5A3	Forward: CGAGTGCCTCTACGTCAGTG Reverse: ATCCATTGGCACTTGGCTCA
AKR1C4	Forward: CTCTCAAGCCAGGTGAGACG Reverse: AGTTTGACACCCCGATGGAC
PDK4	Forward: AGAGGTGGAGCATTTCTCGC Reverse: ATGTTGGCGAGTCTCACAGG

Several studies have shown that cytotoxicity may involve in the cell cycle, checkpoint control and DNA damage responses [51]. To evaluate whether cell cycle arrest was induced by CLs, the cell cycle was analyzed using FCM after treatment with the same concentration and exposure time via PI staining. These data suggest that cell cycle arrest is not obviously involved in the cytotoxicity of CLs in the HepG2 cell line, while the positive control resveratrol triggered retention of HepG2 cells in S-phase.

## Conclusions

**Fig. 6:**
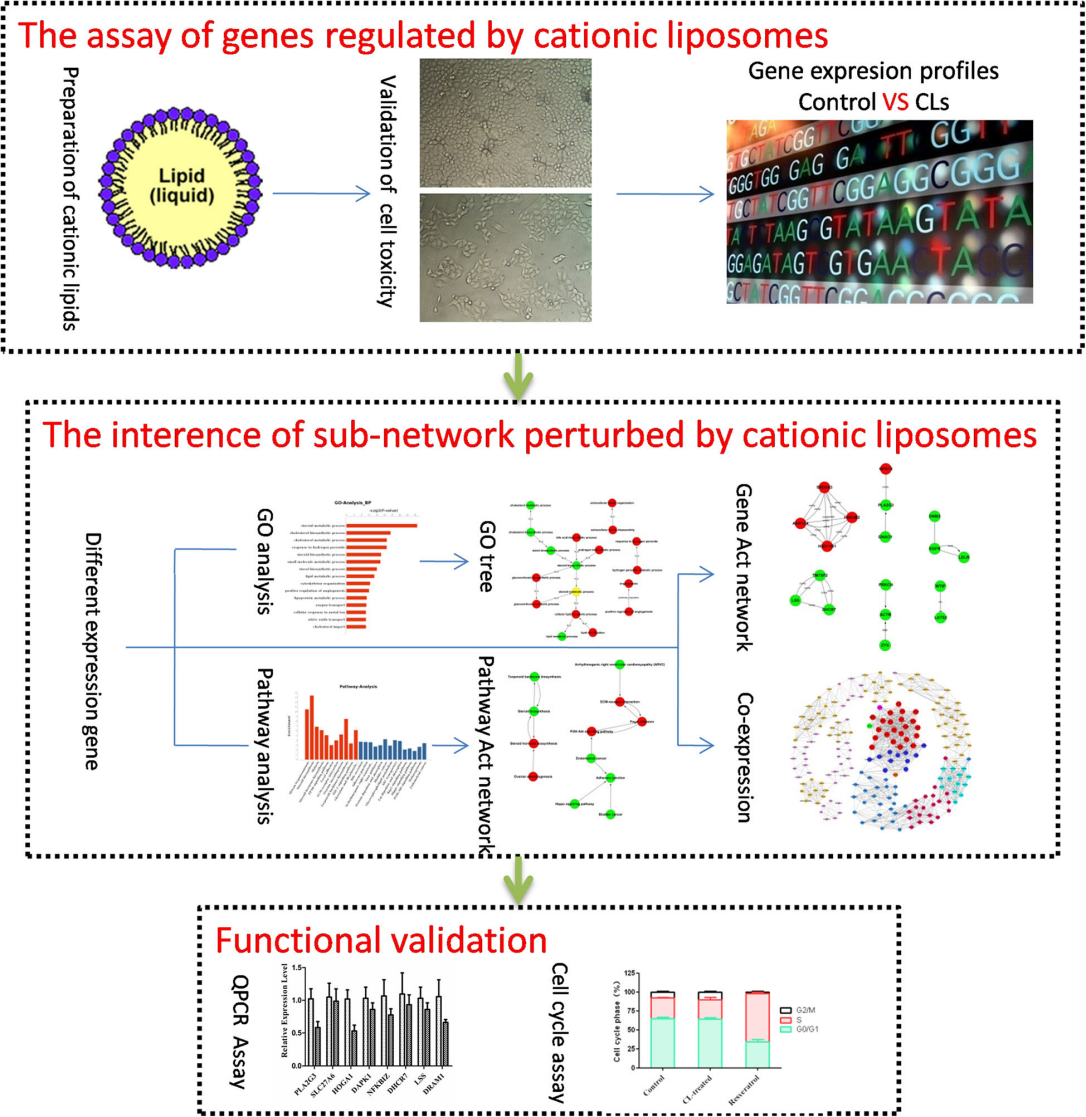
The schematic diagram of this study. The procedure included three steps. Firstly, the cytotoxicity of cationic liposomes (CLs) was detected in HepG2 cell and DEGs in the CLs group comparing with control were identified through next generation RNA-seq technology. Then, functional analysis and bioinformatics computing were employed to explore the key genes and pathways. Finally, expression levels of these genes were confirmed by qPCR, and the cell cycle was assessed by flow cytometry

CLs have been extensively applied for gene and drug delivery as a means of protecting siRNA against enzymatic degradation, facilitating tumor cell uptake, and promoting escape from the endosomal compartment, resulting in effective cytoplasmic delivery. The increasing use of CLs in research and medical products has aroused global concern regarding their fate in biological systems, resulting in a demand for parallel risk assessment. Currently, CLs have only modest success as a delivery vehicle for gene therapy, primarily due to issues with toxicity. The objective of this work was to explore the mechanism of cytotoxicity induced by CLs. According to the previous report [8], the toxicity of cationic liposomes (CLs) is mainly related with their electrical property. Herein, we employed the common used DOTAP CLs in our study. As Fig. 6 shown, we initially successfully synthesized and characterized CLs, and then validated their cytotoxicity in HepG2 cells after 24 h exposure via a CCK-8 assay. The result demonstrated a concentration-dependent cytotoxicity with an IC_50_ of 120 μg/ml. Subsequently, we presented a systems biology approach based on next-generation whole transcriptome sequencing coupled with computational methods, including GO enrichment, pathway and co-expression analysis to reveal key roles involved in cellular responses to CLs. It revealed that cytotoxicity of CLs was mainly related to cholesterol, steroid and lipid biosynthetic and metabolic processes. qRT-PCR was performed to validate the RNA-seq results and FCM indicated the cell cycle arrest was not involved. Fortunately, recent studies have reported that modification to CLs have enabled better, long term transfection capability and low toxicity result in therapeutic efficacy. Our study may provide useful clue for further studies for preventing cytotoxicity. Collectively, CLs could become a promising tool for gene and drug delivery with low toxicity in the future.

## Abbreviations

CLs: Cationic liposomes
DEGs: Differentially expressed genes
FCM: Flow cytometry
GO: Gene ontology
qRT-PCR: quantitative real time PCR
RNA-seq: RNA-sequencing
